# Bayesian spatial and spatio-temporal approaches to modelling dengue fever: a systematic review

**DOI:** 10.1017/S0950268818002807

**Published:** 2018-10-29

**Authors:** A. Aswi, S. M. Cramb, P. Moraga, K. Mengersen

**Affiliations:** 1 ARC Centre of Excellence for Mathematical and Statistical Frontiers, Queensland University of Technology, Brisbane, Australia; 2 Cancer Council Queensland, Brisbane, Australia; 3 Lancaster Medical School, Lancaster University, Lancaster, UK

**Keywords:** Bayesian model, dengue, spatial, spatio-temporal, systematic review

## Abstract

Dengue fever (DF) is one of the world's most disabling mosquito-borne diseases, with a variety of approaches available to model its spatial and temporal dynamics. This paper aims to identify and compare the different spatial and spatio-temporal Bayesian modelling methods that have been applied to DF and examine influential covariates that have been reportedly associated with the risk of DF. A systematic search was performed in December 2017, using Web of Science, Scopus, ScienceDirect, PubMed, ProQuest and Medline (via Ebscohost) electronic databases. The search was restricted to refereed journal articles published in English from January 2000 to November 2017. Thirty-one articles met the inclusion criteria. Using a modified quality assessment tool, the median quality score across studies was 14/16. The most popular Bayesian statistical approach to dengue modelling was a generalised linear mixed model with spatial random effects described by a conditional autoregressive prior. A limited number of studies included spatio-temporal random effects. Temperature and precipitation were shown to often influence the risk of dengue. Developing spatio-temporal random-effect models, considering other priors, using a dataset that covers an extended time period, and investigating other covariates would help to better understand and control DF transmission.

## Introduction

Dengue fever (DF) affects more than 100 million people every year and is one of the most important mosquito-borne diseases in the world [1]. DF is the main source of human deaths from vector-borne disease [2]. The dengue virus is transmitted by a female mosquito of the *Aedes aegypti* species [3, 4] and consists of four serotypes: DENV-1, DENV-2, DENV-3 and DENV-4 [2, 5, 6]. These serotypes can induce a range of symptoms, including the most dangerous stage: dengue haemorrhagic fever (DHF) which is characterised by circulatory collapse and death [4, 7].

In a Bayesian analysis, estimates, predictions and inference are based on posterior distributions. Bayes’ theorem states that this posterior distribution, which expresses the probability of a parameter given the data, equals the multiplication of the likelihood function (the probability of the data given the parameters) with the prior probability distribution for these parameters divided by the probability of the data [8]. This contrasts with a frequentist approach, which derives parameter estimates from the likelihood alone. Bayesian statistical regression models have been used to effectively describe epidemiological data characterised by spatial and spatio-temporal structure [9]. The fundamental feature of Bayesian approaches is the use of probability for measuring uncertainty in inferences [10]. The major appeal of these approaches is in considering uncertainty in the predictions or estimates and the straightforward incorporation of spatial and temporal structure as prior distributions [11]. This approach also allows one to take into account a much wider class of conceptual models than non-Bayesian approaches [12]. The priors can also be used to incorporate information from preceding studies [9].

Previous systematic reviews of DF models have been conducted, but their objectives were different. Three papers have focused on assessing the influence of climate change on transmission of dengue [13–15], one of which specifically considered the effect of temperature [15]. Some reviews have examined the epidemiology of dengue in a certain country, for example, in Thailand [16], Saudi Arabia [17] and four high-income countries [18]. One review reported on different types of modelling methods for early warning systems [1] and the different kinds of spatial methods in dengue transmission [19], respectively.

There has only been one systematic review paper that considered spatial and spatio-temporal modelling approaches to generate a risk map of dengue [20]. This paper identified important predictors for categorical and continuous risks and four types of maps (descriptive, validated, predictive and early warning system). Twelve modelling approaches were identified in 26 publications. The most popular were spatial analyses of case clusters, measures of spatial autocorrelation and logistic regression and multinomial models. However, these methods were not described and Bayesian methods were not discussed.

Despite the appeal of Bayesian models and their popularity in epidemiology, to our knowledge, there are no published systematic review articles of spatial or spatio-temporal modelling of DF using Bayesian methods. The objectives of this systematic review were to identify and review published Bayesian spatial and spatio-temporal models that have been applied to DF, to assess analytical methods including the structure of the model, the use of prior distributions and the inclusion of covariates, and then to identify opportunities for future research.

## Methods

### Search terms and databases

The methodology for this review included a search strategy, and inclusion and exclusion criteria based on the preferred reporting items for systematic reviews and meta analyses (PRISMA) guidelines [21, 22]. Biomedical databases (Medline (via Ebscohost) and PubMed), science databases (ScienceDirect, Scopus and Web of Science) and an all disciplines database (ProQuest) were searched electronically in December 2017. A manual search through reference lists of articles was also undertaken. The literature search was limited to refereed journal articles published from January 2000 to November 2017 in English. Databases were searched with the same keywords, dengue and spati* and Bayesian. The search spati* retrieved spatial, spatio-temporal and spatiotemporal. A Boolean operator was implemented to link the keywords. All results were combined and the duplicates removed using EndNote. The titles and abstracts of articles found through keyword searches were screened first by one author and then the papers identified were evaluated through reading the full text and selected according to the inclusion criteria. This stage was performed by two authors independently. Disagreement between authors was resolved by discussion and consensus.

### Inclusion and exclusion criteria

The inclusion criteria were as follows. First, articles had to be published in a peer-reviewed journal. Second, studies were included if they used Bayesian spatial models or Bayesian spatio-temporal models to model DF. A spatial model was defined as one that explicitly included a geographic index for areas or observations and that then linked these areas in some manner, such as through a random-effects term. Similarly, a temporal model was defined as one that explicitly included a time index. Only English articles were included. No geographical restrictions were applied. The exclusion criteria were as follows: models that were not applied to dengue, non-spatial, non-Bayesian models, modelling of dengue vectors, dengue virus phenotypes, review papers and conference/workshop proceedings. Bayesian models that only considered a temporal component were also excluded. Modelling of dengue mosquito vectors and their egg numbers [23], rather than cases of DF, were excluded. Similarly, modelling the dengue virus was excluded if it was generally about the spread of the dengue virus (via infected humans or mosquitos) and the occurrence of viral genetic diversity. Review papers were read and pertinent studies included, but not the review paper itself. This systematic review is registered on PROSPERO (reference: CRD42018084054).

### Quality assessment

All papers fulfilling the inclusion criteria were critically appraised by two reviewers independently to identify the strengths and weaknesses of each paper. Any disagreement between reviewers was resolved by consensus. The critical appraisal was performed using the adapted tool for assessment of modelling study quality and risk of bias by Harris *et al*. [24] which is a modification of that proposed by Fone *et al*. [25] (Supplementary Table S1). Part A assesses screening questions, Part B checks model validity and Part C assesses the overall results and study conclusions. The adapted tool contains questions for each of eight criteria and clear guidance for scoring. Papers were scored from 0 (poor) to 2 (good) on each of the eight criteria, giving a maximum score of 16 points. A quality level of ‘low’ (<8), ‘medium’ (8–10), ‘high’ (11–13) or ‘very high’ (>13) was assigned to each paper based on the overall score.

### Data extraction

All data were extracted and collected manually. Extracted data included first author, year of publication, study area, time period of study, dengue data (number of cases, time interval collection period and number of areas), covariate data, objectives, analytical method, model structure, key findings, further studies and software. Details of covariates used in the included papers were also extracted.

## Results

### Literature search

**Fig. 1.**
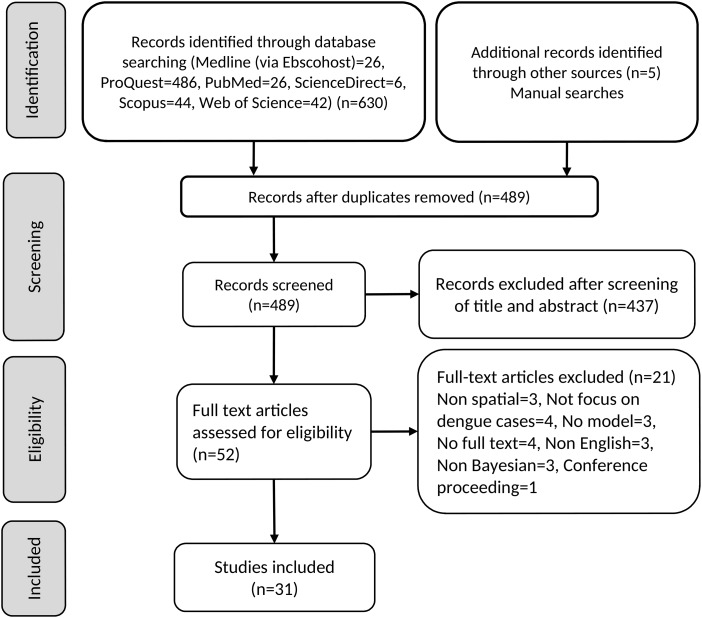
Flow chart of literature search.

The flow chart of this literature search is given in Figure 1, and the list of detailed content of studies is given in Supplementary Table S2. Based on keyword searches, 26 articles from Medline (via Ebscohost), 486 from ProQuest, 26 from PubMed, six from ScienceDirect, 44 from Scopus and 42 from Web of Science were obtained. Five additional records were identified through manual searches. From the 635 citations initially identified, 489 potential relevant articles remained after removal of duplicates (146 duplicate articles). Screening of titles and abstracts removed an additional 437 papers. A further 21 of the 52 remaining articles were excluded for not meeting the inclusion criteria after reviewing the full article. As a result, 31 articles were finally included in the review.

### Dengue data

#### Time intervals and geographic regions

In this review, one study used daily reported dengue cases, eight studies used weekly dengue cases, 15 studies used monthly cases of dengue, one study used quarterly data and six studies used annual dengue cases. The longest period of study was for 384 months (32 years), while the shortest period of study was for 3 months (91 days), with an average of 7 years and a median of 4 years. Eighteen studies used dengue datasets with <7 years, while 13 studies used datasets of 7 years or greater (Supplementary Table S2).

The largest number of districts was 1065 and the smallest number was 10 districts. There were 10 studies in Brazil, six studies in Indonesia, four studies in Taiwan, three studies in Thailand and Australia, respectively, two studies in Colombia and one study in each of Malaysia, China and Puerto Rico.

#### Covariate data

The type and number of covariates included in the models varied widely among the studies reviewed (Table 1). Six categories of covariates were identified, namely climatic, demographic, socio-economic, entomological, geographic and temporal. Although four studies [26–29] examined four of these categories of covariates, most studies used two or three categories while three studies did not include any covariates.

**Table 1.** Covariate variables used in reviewed papers

ID^a^:	1	2	3	4	5	6	7	8	9	10	11	12	13	14	15	16	17	18	19	20	21	22	23	24	25	26	27	28	29	30	31	∑
Climatology																																
Temperature		✓						✓		✓		✓	✓	✓	✓	✓	✓	✓				✓				✓	✓	✓		✓		15
Precipitation	✓	✓		✓				✓	✓	✓		✓	✓	✓	✓	✓			✓			✓		✓		✓	✓	✓		✓		18
El Niño Southern Oscillation Index													✓															✓				2
Oceanic Niño Index														✓																		1
Demography																																
Population density							✓		✓		✓			✓	✓				✓					✓	✓						✓	9
Proportion of overseas visitor								✓																								1
Age structure			✓								✓																✓					3
Percentage of urban population													✓																			1
The mean age of population											✓																					1
Household density											✓																					1
Human daily mobility																															✓	1
Ratio of male and female																									✓							1
Socio-economic																																
Income			✓		✓	✓		✓			✓																					5
Garbage collection			✓		✓	✓					✓														✓							5
Water supply					✓	✓					✓														✓							4
Literacy			✓		✓	✓		✓			✓														✓		✓					7
Occupation								✓																			✓					2
Living condition (slums)					✓			✓																								2
Sewage disposal			✓								✓																					2
Mean number of people per household											✓																					1
Percentage of black people											✓																					1
District's Index of Human Development					✓																											1
Entomology																																
Breteau index																												✓			✓	2
Larva -Free Home Index									✓																							1
Healthy Housing Index									✓																✓							2
Indoor residual spraying (IRS)																										✓						1
Mosquito density																					✓											1
Geography																																
Altitude													✓	✓	✓																	3
Mean vegetation index																	✓	✓									✓					3
Mean elevation (m)											✓																					1
Elevation range (m)											✓																					1
Distance from census tracts to public emergency health unit											✓																					1
Longitude							✓																									1
Latitude							✓																									1
Percentage of area covered by mountain					✓																											1
Location of strategical points for breeding of *Aedes aegypti*					✓																											1
Temporal																																
Covariate -specific distributed lags		✓								✓			✓	✓	✓	✓					✓							✓				8
Year																						✓										1
Non -linear temporal trend																										✓						1

a Refers to numbers in Table 2.

**Table 2.** Summary of the structure of the spatio-temporal models discussed in the reviewed paper

ID^a^	References	Year	Space	Time	Space–time
1	Astutik *et al*. [34]	2013	–	–	SAR^a^
2	Chien and Yu [49]	2014	CAR^b^	Cubic spline	–
3	Costa *et al*. [41]	2013	CAR	–	–
4	Fernandes *et al*. [33]	2009	–	–	CAR
5	Ferreira and Schmidt [43]	2006	CAR	–	–
6	Honorato *et al*. [39]	2014	CAR	–	–
7	Hu *et al*. [51]	2011	CAR	–	–
8	Hu *et al*. [40]	2012	CAR	–	–
9	Jaya *et al*. [36]	2016	CAR	–	–
10	Johansson *et al*. [32]	2009	Normal	CSS^c^	–
11	Kikuti *et al*. [38]	2015	CAR	–	–
12	Lekdee and Ingsrisawang [52]	2013	CAR	–	–
13	Lowe *et al*. [26]	2011	CAR	AR(1)^d^	–
14	Lowe *et al*. [27]	2013	CAR	AR(1)	–
15	Lowe *et al*. [28]	2014	CAR	AR(1)	AR(1)
16	Lowe *et al*. [48]	2016	CAR	AR(1)	–
17	Martínez-Bello *et al*. [47]	2018	Leroux CAR	RW1^e^	Normal
18	Martínez-Bello *et al*. [46]	2017	Leroux and BYM^f^ CAR	–	–
19	Mukhsar *et al*. [37]	2016a	–	Temporal trend	CAR
20	Mukhsar *et al*. [53]	2016b	–	Temporal trend	CAR
21	Pepin *et al*. [44]	2015	Gravity model	–	–
22	Restrepo *et al*. [50]	2014	CAR	–	–
23	Samat and Percy [54]	2012	CAR	–	–
24	Sani *et al*. [35]	2015	–	Temporal trend	CAR
25	Vargas *et al*. [42]	2015	Kernel quartic function	–	–
26	Vazquez-Prokopec *et al*. [31]	2010	Markov random field	P-splines^g^	–
27	Wijayanti *et al*. [29]	2016	Normal	Normal	Normal
28	Yu *et al*. [30]	2011	–	–	BME^h^
29	Yu *et al*. [55]	2014	–	–	BME–SIR
30	Yu *et al*. [56]	2016	–	–	BME
31	Zhu *et al*. [45]	2016	Normal	–	–

a Spatial autoregressive (SAR).

b Conditional autoregressive (CAR).

c Cubic spline smoothing (CSS).

d First-order autoregressive (AR(1)).

e First-order random walk (RW1).

f Besag–York–Mollié (BYM).

g Penalised splines (P-splines).

h Bayesian Maximum Entropy (BME).

#### Climatic covariates

More than half of the studies (20 out of 31) used climatic variables in modelling DF disease. The most commonly used predictors included temperature and precipitation. Two studies additionally included the El Niño Southern Oscillation Index (SOI) [26, 30]. One study used temperature, precipitation and Oceanic Niño Index (ONI) [27]. Mean temperature, minimum temperature and maximum temperature [31], night-time temperature and day-time temperature [29] and monthly mean maximum temperature, mean minimum temperature and cumulative precipitation [32] were also included as climatic predictors in the reviewed models. Five studies used precipitation only [33–37].

#### Demographic covariates

Out of 31 studies, 13 included demographic data. Most studies only used one of the eight categories of demographic variables considered in Table 1, while only one study used four categories: population density, age structure, mean age of population and household density [38]. Population density was the most common demographic variable used in modelling DF.

#### Socio-economic covariates

Socio-economic data were used in seven studies [29, 38–43]. The most common socio-economic variable was educational level (seven studies), followed by income and garbage collection (five studies each). One study used seven categories of socio-economic variables [38].

#### Entomological covariates

Only six studies incorporated entomological (mosquito) data in their models [30, 31, 36, 42, 44, 45]. A Breteau index (BI) which is defined as the number of positive containers (i.e. containing *A. aegypti* larvae) per 100 houses inspected, was used as a predictor in two studies [30, 45] and a House Index (HI), which is defined as percentage of houses infested with larvae and/or pupae, was used as a predictor in another two studies [36, 42] to identify dengue transmission areas. A larva-free home index and a healthy housing index were included to determine their impact on the DF relative risk [36]. Indoor residual spraying has also been considered in modelling DF [31].

#### Geographic covariates

Nine studies used geographic characteristics in their model. Altitude [26–28] and mean vegetation index [29, 46, 47] were the most common features used. Out of nine geographic variables applied, most studies used only one indicator. Only one study [38] used three indicators, which were the mean elevation to sea level, elevation range and distance from the census tracts (CTs) centroid to the health service.

#### Temporal covariates

Temporal data were used in 10 studies. Most studies included a temporal lag in the climate data [26–28, 30, 32, 44, 48, 49] and only two studies included temporal entomological data [30, 44]. Models have included time in years as a categorical variable to evaluate the dynamics of dengue cases [50] and also non-linear temporal trends [31].

### Analytical method

A variety of Bayesian spatial and spatio-temporal approaches were used in modelling DF. Most studies adopted a fully Bayesian model with a spatially structured random effect using a CAR prior structure to investigate the relationship between the risk of dengue and selected covariates [36, 38–41, 43, 46]. Spatial empirical Bayes smoothing was used for two studies to examine the spatial distribution of dengue [42, 51].

Generalised linear mixed models (GLMMs) with proper CAR spatial random effects were applied to develop disease maps, with dengue incidence data assumed to be Poisson [52]. Temporal components were additionally incorporated, either as a temporal covariate [44, 50], or via a GLMM with spatial and temporal random effects and temporal covariates [26, 27, 48]. Among the selected studies, only two studies used a GLMM with spatial, temporal and spatio-temporal random effects [29, 47], while one included these components along with an additional temporal covariate [28]. Other GLMM spatio-temporal random-effects models with incorporation of a temporal trend have also been developed [35, 37]. Two studies used a GLMM zero-inflated model [33, 53].

Alternative models included estimation of relative risk for the transmission of dengue disease based on discrete time and space via a susceptible–infective-recovered model for human populations; susceptible–infective model for mosquito populations (SIR-SI) [54], prediction of spread of DF using Bayesian maximum entropy (BME) [30, 55, 56], and spatio-temporal quasi-Poisson model based on a DLNM (distributed lag non-linear model) [49], STARM (spatial–temporal autologistic regression model) [34], hierarchical model with adaptive natural cubic spline [32], a semi-parametric Bayesian STAR (structured additive regression) model [31] and a transmission model based on Ross–Macdonald theory [45]. The analytical methods used across all included studies are summarised in Supplementary Table S3 and summary of the structure of the spatio-temporal models discussed in the reviewed paper can be seen in Table 2. These models are explained in more detail as follows.

#### Spatial models

Several spatial models have been developed and applied to DF, namely empirical Bayes approaches and fully Bayes GLMM with a spatial CAR prior.

*Empirical Bayes approaches:* An empirical Bayes method is an approximation to the fully Bayesian method. In an empirical Bayes approach, the prior parameters are estimated from the data, while in a fully Bayesian analysis, the prior distribution is completely specified before observing any data [57].

Empirical Bayes spatial smoothing for dengue incidence data has been used to categorise the high-risk and low-risk areas in Queensland [51]. Local empirical Bayes was applied to investigate the relationship between the HI, dengue incidence and socio-demographic variables [42]. The authors concluded that there is a positive correlation between HI and Bayesian dengue incidence rate. The highest dengue risk regions were situated in the areas which had the highest population densities and were close to the major highways.

*GLMM with spatial random effects:* A GLMM with spatial random effects has been applied in seven studies [36, 38–41, 43, 46]. The general model is formulated as follows:

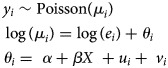

where *y*_*i*_ is the number of dengue cases in *i* = 1, …, *I* areas; *e*_*i*_ and *θ*_*i*_ are, respectively, the expected number of dengue cases in area *i* and the log relative risk of dengue; *α* is the overall level of relative risk; *β* = (*β*_1_, *β*_2_, …*β*_*p*_) represent the coefficient of the covariates; *u*_*i*_ is a spatially structured random effect with CAR prior structure and *v*_*i*_ is a spatially unstructured random effect with mean zero and variance 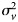. All authors used an intrinsic Gaussian CAR (ICAR) prior and adopted a binary neighbourhood weighting. An ICAR model assumes the areas *k* and *i* are neighbours if both share a common border. This can be expressed as follows:
1
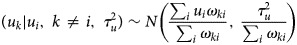


*ω*_*ki*_ = 1 if *k, i* are adjacent, *ω*_*ki*_ = 0 otherwise [58, 59]. This prior is the most common Gaussian Markov random field [60] and is an improper prior [61]. Allowing for spatial autocorrelation through this prior can improve model fit [38]. However, the choice of neighbourhood structure needs to be carefully considered as it could impact on the significance of some covariates [43]. One study examined two additional types of neighbourhood structure matrices, namely, weighted by the length of the boundary and by boundary and barriers [43].

Martínez-Bello *et al*. [46] compared CAR BYM (Besag, York and Mollié) [62] prior and Leroux CAR prior [63] for spatially structured random effects for estimating relative risk of dengue. They found that the CAR BYM prior was better than the Leroux CAR prior.

### Spatio-temporal models

*GLMM over space and time with spatial random effects:* A GLMM indexed by space and time and with spatial random effects has been proposed in one study [52] to develop a disease map and identify any association between dengue incidence, rainfall and temperature. The proposed model is expressed as

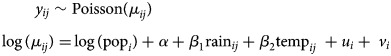

where *y*_*ij*_ are the number of dengue cases in area *i* = 1, …, *I*, and time *j* = 1, …, *J*; *μ*_*ij*_ is mean cases, log(pop_*i*_) is the offset representing the total population in each area. Rain and temp are the total rainfall and temperature, respectively, in each area and time, and *u*_*i*_ are proper CAR spatial random effects. A proper CAR is a variant of the ICAR prior, with an additional term for spatial autocorrelation *ρ* in the conditional expectation [64], as follows:

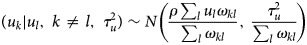


If *ρ* = 1 then the model is the ICAR in equation (1).

*GLMM with spatial random effects* *+* *temporal covariate:* An alternative representation of a GLMM with spatial random effects and the inclusion of temporal covariates has been proposed in two studies [44, 50]. Restrepo *et al*. [50] found that the convolution model was the preferred model (this includes both *u*_*i*_ and *v*_*i*_) over models containing only the uncorrelated term*v*_*i*_ or the ICAR term *u*_*i*_, and precipitation was the most significant predictor of dengue risk.

Pepin *et al*. [44] proposed a different GLMM formulation to assess the role of city-wide vector data in forecasting DF cases. In this model, they included the rate of cases in neighbourhood *i* at time *j*, mosquito density data, fixed scaling factors, lagged time for specific variables and different weighting functions between-neighbourhood effects to illustrate patterns of city-wide human movement which consists of economic value of the neighbourhood, population density and travel distance between neighbourhoods. Two scales of spatial disease data, that is, nearest-neighbourhood effects (local) and all between-neighbourhood effects (global) are compared to predict the association between mosquito density and human cases of dengue. Models that included global between-neighbourhood effects and two covariates (mosquito density and human cases of dengue) and their interaction were preferred.

*GLMM with spatial and temporal random effects* *+* *temporal covariate:* GLMMs with spatial and temporal random effects and a temporal covariate have been proposed in three studies [26, 27, 48]. Lowe *et al*. [26] compared a spatio-temporal GLM and a GLMM that includes random effects in the linear predictor and found that the latter model provided more accurate dengue predictions. In this model, the number of dengue cases *y*_*ij*_ are assumed to be Poisson distributed with mean dengue count *μ*_*ij*_ given by

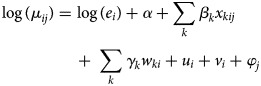

where 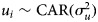, 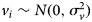, and *φ*_*j*_ are the temporally autocorrelated random effects (*j* = 2, …, 12) with *φ*_1_ = 0, and 
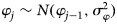
, *j* = 2, …, 12.

The variable climate factors *x*_*kij*_ are: precipitation in the previous 1 and 2 months, temperature in the previous 1 and 2 months and Nino 3.4 in the previous 6 months. The variables *w*_*ki*_ are: altitude and percentage of urban population.

Another spatio-temporal GLMM by Lowe *et al*. [27] extended the model by Lowe *et al*. [26] by adding more recent data and including log dengue standardised morbidity ratio in the previous 3 months (past dengue risk), spatially structured and unstructured random effects and a first-order autoregressive month effect. Here the DF counts *y*_*ij*_ are assumed to have a negative binomial distribution to allow for overdispersion in observed dengue data. The authors compared this model with a simple model based on past dengue risk only. They found that the extended model improved dengue predictions.

Generalised linear and additive mixed models (GLMM/GAMM) were applied to measure the benefit of including climate function in the model [48]. The response had a negative binomial distribution and the dengue relative risk models included a baseline model with season only, a seasonal–spatial model (inclusion of spatial structure and unstructured error), a seasonal–spatial climate-linear model (linear climate model) and a seasonal–spatial climate-non-linear model (non-linear climate model). The results showed that the model with linear and non-linear climatic functions explained 39% and 40%, respectively, of the variation in dengue relative risk. An additional 7% and 8% of the variation was explained by seasonal–spatial structure using linear and non-linear climatic functions, respectively.

*GLMM with spatial, temporal and spatio-temporal random effects* *+* *temporal covariate:* Lowe *et al*. [28] also formulated another GLMM model using a negative binomial distribution for the dengue case counts to predict dengue epidemic in Brazil during the 2014 football tournament. This model has minor differences and extensions to their previous model [27]: the inclusion of log dengue standardised morbidity ratio 4 months previously, a fixed effect for month, a random effect for month and the inclusion of a first-order autoregressive month effect for each zone. Their results showed that this model can forecast which cities have low, medium and high risk of dengue.

*GLMM with spatial, temporal and spatio-temporal random effects:* Bayesian spatial, temporal and spatio-temporal random-effects models have been used to determine factors that influence the risk of dengue in the Banyumas regency, Indonesia [29]. Two models have been compared, namely a model with the inclusion of covariates and spatially structured random effects only and a model with the inclusion of covariates, spatially structured and unstructured random effects, temporally structured and unstructured random effects and spatio-temporal random effects. The number of DF cases was assumed to be Poisson distributed. Uninformative priors were used for all variables as previous data were not available for Indonesia. The most significant factors that influenced the risk of dengue were found to be employment type and economic status. Wijayanti *et al*. [29] explored only the unstructured interaction effect model (type I). The type II–IV interaction effects in spatio-temporal models of relative risk were not explored, which are temporal interactions, spatial interactions and inseparable space–time interactions, respectively. Martínez-Bello *et al*. [47] explored type I–IV interaction effects, finding that the best model had the inclusion of a fixed coefficient of lag-zero epidemiological periods Land Surface Temperature (LST) and type IV interaction effects.

*GLMM with spatio-temporal random effects* *+* *temporal trend:* Sani *et al*. [35] developed a spatio-temporal convolution model as an extension of the spatial convolution model introduced by Eckert *et al*. [65] and used this to analyse the relationship between covariates (rainfall and population density) and dengue risk. The number of dengue cases *y*_*ij*_ was assumed to be Poisson distributed and the relative risk *μ*_*ij*_ given by:

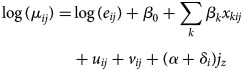

where (*α* + *δ*_*i*_)*j*_*z*_ is a temporal trend and

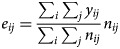

with *n*_*ij*_ denoting the number of population at area *i* time *j*.

The authors found that both rainfall and population density affected the number of dengue cases.

This spatio-temporal convolution model has been extended to include the probability of incident risk 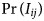 into the model to overcome a misidentification of dengue location [37]. The extended model is as follows:
2
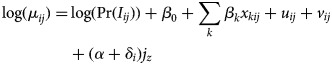

where

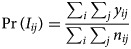


This extension resulted in more accurate estimates when compared with the previous models [35, 65]. They also concluded that both rainfall and population density significantly affected the number of dengue cases.

*GLMM zero-inflated Poisson spatio-temporal model:* Zero-inflated spatio-temporal models that can be applied to both continuous and discrete data have been proposed [33]. When observations exhibit an excessive number of zero values, the zero-inflated model is often more appropriate. These have been applied to estimate the probability of the presence of unobserved dengue disease in region *i* and time *j*.

A Bayesian mixed zero-inflated Poisson spatio-temporal (BMZIP S-T) model [53] has also been constructed.

The BMZIP S-T model is expressed as

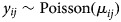

where *μ*_*ij*_ = *ϕ*_*ij*_/(1 − *ϕ*_*ij*_) and is modelled as per equation (2).

*A spatio-temporal quasi-Poisson model:* A spatio-temporal quasi-Poisson model based on the DLNM approach has been proposed to identify the relationship between the non-linear delayed impact of meteorological variations and dengue risk in southern Taiwan and to predict dengue cases in the coming weeks [49]. The number of weekly DF cases *y*_*ij*_ was assumed to have a Poisson distribution as follows.

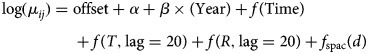

where the vector *β* contains the coefficients of the indicator variable year, *f*(Time) is the time smoother described by a cubic spline; *f*(*T*, lag = 20) and *f*(*R*, lag = 20) are functions of temperature and rainfall with a maximum temporal lag of 20 weeks, respectively; *f*_spac_(*d*) is a spatial function which was modelled using the CAR prior structure, and the offset is the logarithm of average annual population data. The authors found that the most significant factors that influenced DF epidemics were the weekly minimum temperature and the maximum 24 h rainfall. When the minimum temperature rises, the dengue relative risk increases, particularly at a lagged period of 5–18 weeks.

*Hierarchical model with adaptive natural cubic spline:* Johansson *et al*. [32] proposed a model that includes population size *N*_*j*_, covariates at distributed lags *l*_*k*_ and a natural cubic spline smoothing function of time *s*(*j*, *λ*), where *λ* denotes the degree of annual freedom and is set to *λ* = 2. The distributed lag model is used to evaluate the effect of weather on dengue spread in the next 6 months. For each area *i*, the number of monthly dengue cases at time *j*, *y*_*j*_, is assumed to be Poisson distributed as follows:

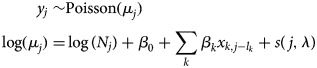


A two-level approach was used to compare *β*_*k*_ from the area-specific models. At the first level, area-specific (*i*) parameter estimates 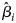 were assumed to be normally distributed:

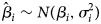


Effect modifiers *z*_1_, *z*_2_, …, *z*_*Q*_ were added to estimate *α*_0_ (the average effects) and the effect modification *α*_*q*_:

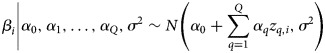


The authors found a positive correlation between monthly variation in temperature and precipitation and monthly variation in the spread of dengue, and that correlation varies spatially.

*BME method:* BME is popular in the study of natural systems (physical, biological, social or cultural) and for attributes that are characterised by space–time dependence and multi-sourced uncertainty. Two major knowledge bases (KB) for the spatio-temporal modelling in the BME method are: (1) the general KB (G-KB) that may include scientific theories, theoretical space–time dependence models and epidemic models; and (2) the site-specific KB (S-KB) that includes hard data and soft data, often with a significant amount of uncertainty [66]. The BME method incorporates both knowledge bases [67, 68].

A spatio-temporal model that is based on a stochastic BME method has been used to predict DF outbreaks based on space and time and to examine the association between DF incidence and selected climate variables in Southern Taiwan [30]. In the BME analysis, the spatio-temporal distribution of DF occurrences is mathematically represented by the spatio-temporal random field, *X(p)* or *X*_*i,j*_ where *i* and *j* indicate the areas and time, respectively. DF incidence is assumed to be Poisson distributed as follows:

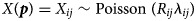

with DF mean 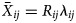 and *λ*_*ij*_ is a climate-driven space–time process modelled by the log-link Poisson regression

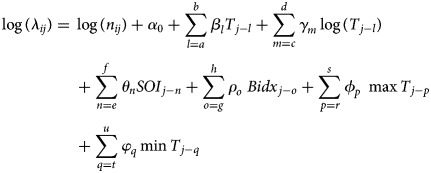

where *β*_*l*_, *γ*_*m*_, *θ*_*n*_, *ρ*_*o*_, *ϕ*_*p*_ and *φ*_*q*_ are regression coefficients for temperature, logarithm of rainfall, SOI, BI, maximum temperature and minimum temperature, respectively (for the weekly temporal lags between *a* and *b*, *c* and *d*, *e* and *f*, *g* and *h*, *r* and *s*, and *t* and *u*, respectively) and *n*_*ij*_ is the population size. The authors conclude that climatic conditions significantly affect DF outbreaks. Yu *et al*. [55] extended their previous model by inclusion of a stochastic susceptible–infected–recovered (SIR) model, that is, BME-SIR to obtain online space–time predictions of DF transmission. This model considered stochastic differential equations, characterising both the spatio-temporal pattern of disease spread and the heteroscedastic variance pattern across space and time. The aim was to achieve online updates of SIR model parameters.

*A SIR-SI model:* A discrete space–time stochastic susceptible–infective–recovered for human populations; susceptible–infective for mosquito populations (SIR-SI) model has been developed to circumvent problems of relative risk estimation using standardised morbidity ratios and the Poison-*γ* model which does not allow for spatial correlation [54]. The SIR-SI model was defined as follows:

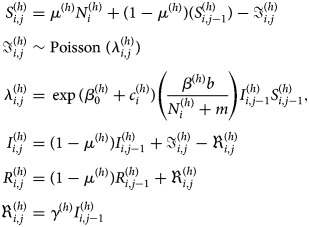


with a non-stochastic vector population as follows:

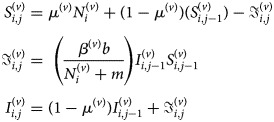


Here the superscripts (*h*) and (*v*) represent the human and mosquito populations, respectively. 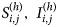 and 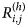 are the total number of susceptible, infective and recovered humans in area *i* for time *j*, respectively; 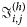 and 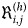 are the number of newly infective and recovered humans; *μ*^(*h*)^ is the weekly birth and death rates in the human population; *γ*^(*h*)^ is the rate of weekly recoveries; *b* is weekly biting rate; *m* is the number of alternative hosts available; *β*^(*h*)^ is the probability of transmission from mosquito to human, and *β*^(*v*)^ is the converse; and 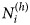 is the number of humans in area *i*.

The number of new infections is assumed to follow a Poisson distribution with mean 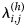, intercept 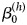 and spatial random effect 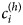 using a CAR prior. Models were applied to all of Malaysia divided into 16 states. The results showed that the proposed SIR-SI model that considers the inclusion of the transmission process of dengue disease, covariates and spatial correlation was preferred over unmodelled SMRs or the Poisson-*γ* model. The authors also identified areas with very high and high dengue risk.

*STARM:* The STARM model is an extension of an autologistic regression model that includes covariates, spatial and temporal dependence simultaneously. This model has been applied to predict the association between the incidence of endemic dengue and rainfall using a Bayesian method [34]. For binary data that are measured repeatedly on a spatial lattice, STARM can be very beneficial [69]. The incident rate (IR) is converted to the binary scale as a representation of the *A. aegypti* spread, that is, 1 if there is endemic dengue (IR > 10/100 000 population) and 0 if there is no endemic dengue. Endemic level and rainfall are dependent and independent variables, respectively. The STARM model may be defined as follows:

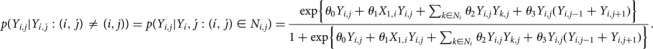

where *Y*_*i*,*j*_ is dengue endemic at the *i*th region and the *j*th time, *X*_1,*i*_ is rainfall index at the *i*th region, *N*_*i*,*j*_ is neighbourhood structure. *θ*_0_, *θ*_1_, *θ*_2_, *θ*_3_ are an intercept and coefficients for rainfall, spatial autoregression and temporal autoregression, respectively. The authors use the inverse Gaussian as a prior distribution for each of *θ*_0_, *θ*_1_, *θ*_2_, *θ*_3_. The result showed that there is a positive correlation between the endemic level of DHF incidence and rainfall.

*A semi-parametric Bayesian spatio-temporal geoadditive STAR model:* A spatio-temporal geoadditive STAR model has been used to evaluate the impact of indoor residual spraying and spatial autocorrelation in the odds of dengue infection [31]. A predictor structure for the spatio-temporal geoadditive model is given as follows:

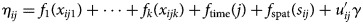

where *η*_*ij*_, *x*_*ij*1_, …, *x*_*ijk*_ are predictor and covariate values for individual *i* at time *j*. The fixed effects of non-linear function of covariates (*f*_1_, …, *f*_*k*_) and non-linear time trend *f*_time_ were modelled by independent diffuse priors using Bayesian penalised splines. *f*_spat_ is a spatially structured random effect of the location *s*_*ij*_ using Markov random field priors and 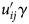 are linear predictors for the covariate vector *u*.

This STAR model assessed the impact of rain, spray cumulative proportion (cum_spr) and spatial correlation (spat) on the odds of dengue virus infection, where the probability of infection followed a binomial distribution (0 if there is no infection, 1 if there is an infection) at house level (1490 premises) as follows:

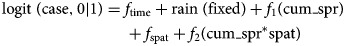


The authors compared two STAR models, that is, a model with and without rain as a fixed effect. Interestingly the results showed that a model without a rain covariate was better able to describe the spatial pattern of dengue infection. The authors concluded that there was a significant positive correlation between the number of indoor residual spraying applications up to a time lag of 2 weeks and the weekly number of cases.

*Transmission model based on Ross–Macdonald theory:* A dengue transmission model based on the Ross–Macdonald theory has been proposed to identify the pattern of dengue transmission in space and time. This model incorporates four essential sub-models, that is, female mosquito density dynamics, human daily movement, virus transmission and estimation of parameters [45] that can be explained as follows.

The correspondence between reported incidence and modelling cases is given as follows:

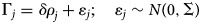

where *δ* is reported incidence rate, *ρ*_*j*_ = (*ρ*_1*j*_, *ρ*_2*j*_, …, *ρ*_*Ij*_)^*J*^ is a vector of the estimated number of incidences at each time period *j*, *ε*_*j*_ is an error term and Γ_*j*_ = (*γ*_1*j*_, *γ*_2*j*_, …, *γ*_*Ij*_)^*J*^ is the vector space–time surveillance data at time *j*.

Female mosquito density 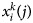 with age *k* at time *j* in district *i* can be calculated as

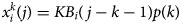

where *K* is the proportionality coefficient between the vector density and BI, *B*_*i*_(*j*) is the value of BI at time *j* in district *i*, *p(k)* is the daily survival probability of adult mosquitoes at age *k*. To estimate model parameter *K*, MCMC methods were used.

Human daily commuting into different areas, which is defined as those who work or study in different districts and who go out in the morning and return in the afternoon, is assumed to impact on dengue transmission as follows:

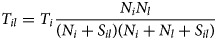

where *T*_*il*_ is the number of travellers leaving from district *i* to *l*; *N*_*i*_ is the population in district *i*; *S*_*il*_ is the total number of residents in the circle whose centre is the origin district *i* and radius is the distance between district *i* and the destination district *l*, minus the population at *i* and *l. T*_*i*_ is the total number of travellers leaving from district *i* which is defined as *T*_*i*_ = *N*_*i*_(*N*_*c*_/*N*), where *N*_*c*_ and *N* are the total number of travellers and the total population, respectively.

In virus transmission modelling, vectorial capacity, which is defined as the mean of infectious mosquito bites per unit time, is used to evaluate the infectivity from mosquitoes and is calculated as

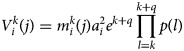

where 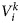, 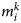 represent vectorial capacity contributed by mosquitoes with age *k* in district *i*, and the ratio of mosquitoes at age *k* to humans, respectively. Here *a*_*i*_ and *e*^*k*+*q*^ represent *Aedes* mosquito biting rate of humans in district *i* within 12 h, and the expectation of remaining infectious life at age *k* *+* *q*, respectively.

The authors concluded that the space–time distribution of incidence is highly heterogeneous, with 81.6% of transmission occurring in urban centres in Guangzhou, China with a peak in mid-October. They also found that there is inconsistency between infected cases and reported cases in space–time. Vector indices and human mobility factors significantly affect the dengue transmission patterns. Urban areas had the highest incidence rates and suburban areas had the second highest incidence rates.

### Assessment of quality

Using the adapted tool for assessment of modelling study quality, quality scores for the reviewed paper ranged from 7 to 16 out of 16 (Table 3). One study was classified as low quality, three as medium quality, 10 as high quality and 17 as very high quality. The median score was 14/16, which is categorised as high quality. Details on the quality of data were lacking in many papers.

**Table 3.** Assessment of included modelling studies

No	Author	Year	AaO	SaP	MS	MM	PRDS	QoD	PoR	IDoR	FS	Rating
1	Astutik	2013	2	1	2	2	2	0	1	1	11	High
2	Chien	2014	2	2	2	2	1	2	2	2	15	Very high
3	Costa	2013	2	2	2	2	1	2	2	2	15	Very high
4	Fernandes	2009	2	1	2	2	2	0	2	2	13	High
5	Ferreira	2006	2	2	2	2	1	2	2	2	15	Very high
6	Honorato	2014	2	2	2	2	1	2	2	2	15	Very high
7	Hu	2011	2	1	1	1	1	2	2	2	12	High
8	Hu	2012	2	1	2	2	2	2	2	2	15	Very high
9	Jaya	2016	1	2	2	2	1	0	2	1	11	High
10	Johansson	2009	1	1	1	1	1	0	2	1	8	Medium
11	Kikuti	2015	2	2	1	2	2	2	2	2	15	Very high
12	Lekdee	2013	2	1	2	2	1	0	2	1	11	High
13	Lowe	2011	2	1	2	2	2	2	2	2	15	Very high
14	Lowe	2013	2	1	2	2	2	2	2	2	15	Very high
15	Lowe	2014	2	1	2	2	1	2	2	2	14	Very high
16	Lowe	2016	2	2	2	2	2	2	2	2	16	Very high
17	Martínez-Bello	2018	2	2	2	2	2	2	2	2	16	Very high
18	Martínez-Bello	2017	2	2	2	2	2	2	2	2	16	Very high
19	Mukhsar	2016a	1	1	1	1	1	1	1	1	8	Medium
20	Mukhsar	2016b	0	2	1	1	1	0	1	1	7	Low
21	Pepin	2015	2	1	2	2	1	2	2	2	14	Very high
22	Restrepo	2014	2	1	2	2	2	2	2	2	15	Very high
23	Samat	2012	2	1	1	2	1	0	1	1	9	Medium
24	Sani	2015	2	1	1	2	1	0	2	2	11	High
25	Vargas	2015	2	2	0	1	1	2	1	2	11	High
26	Vazquez-Prokopec	2010	2	2	2	2	2	2	2	2	16	Very high
27	Wijayanti	2016	2	1	2	2	2	1	2	2	14	Very high
28	Yu	2011	2	1	1	1	1	1	2	2	11	High
29	Yu	2014	2	1	1	2	1	0	2	2	11	High
30	Yu	2016	2	1	1	2	1	1	1	2	11	High
31	Zhu	2016	2	1	2	2	2	2	2	2	15	Very high
Median score			2	1	2	2	1	2	2	2	14	Very high
Mean score			1.8	1.4	1.6	1.8	1.4	1.3	1.8	1.8	12.9	High

AaO, aims and objectives; SaP, setting and population; MS, model structure; MM, modelling methods; PRDS, parameter ranges and data sources; QoD, quality of data; PoR, presentation of results; IDoR, interpretation and discussion of results; FS, final score.

## Discussion

### Covariates

Climatic variables were dominant among covariates used by studies to predict the DF outbreaks based on place and time. Precipitation and temperature were the most common and most commonly significant predictors [32, 49, 52]. Most studies found a positive significant association with precipitation [34, 35, 37, 50], although one study found a negative correlation with precipitation 4–6 months previously [48]. These more complex associations depend on local seasonal patterns.

Population density was the most common demographic factor used in modelling DF. Study results were generally consistent in showing that population density was positively significantly correlated with an increase in dengue cases [28, 35, 37, 42]. Furthermore, urban areas had higher incidence rates than suburban areas [45]. Kikuti *et al*. [38] found that population density and the percentage of population aged under 15 years were correlated with detection of dengue. Human daily mobility as an indicator of demography, referring to commuting, was included for only one study [45]. Human movement significantly affects the spatial spread of infectious disease like dengue [70, 71]. Therefore, it is important to incorporate the variety of human movements in modelling dengue transmission.

The most common socio-economic variable was educational level, followed by income and garbage collection. The significance of socio-economic factors differ by regions, but dengue is often more common among those of lower socio-economic status. For example, in Brazil, inadequate garbage disposal and income were the most significant factors related to the incidence of dengue [39] and lower socio-economic status (within a slum society) increased the risk of dengue [38]. In Indonesia, the most significant factors that influenced the risk of dengue were employment type and education level [29].

Incorporation of entomological data in modelling DF to determine their impact on DF relative risks has been used by only six studies. Some studies have found that the most significant effect on the relative risk of DF is a larva-free home [36] and there was a significant positive correlation between the number of indoor residual spraying applications up to a time lag of 2 weeks and the weekly number of cases [31]. Without mosquitoes, dengue cannot be transmitted.

Geographical data were used in nine studies reviewed. The most common indicator was altitude [26–28] and mean vegetation index [29, 46, 47]. Only one study [38] used three indicators, namely the mean elevation from sea level, elevation range and distance from the CTs to the public health unit. The other studies used only one indicator. Lowe *et al*. [28] found that altitude was significantly negatively correlated with relative risk of dengue. Kikuti *et al*. [38] showed that residential adjacency to the health unit was most significantly correlated with dengue cases detection and the spatial distribution of dengue cases detection was heterogeneous. Therefore, it is important to consider neighbourhood features when evaluating DF risk.

This review has also shown that almost all the reviewed papers that include a lag time use climate data [26–28, 30, 32, 44, 48, 49] and only a few papers include entomological data [30, 44]. Various temporal lags and climate variables have been used to find the most significant combination of temporal lags in describing the relative risk of dengue and predicting DF outbreak. For example, in Thailand, temperature and precipitation were significant dengue predictors with a time lag of 1 month preceding, but precipitation 4–6 months preceding was negatively correlated with dengue relative risk [48]. Three studies reported on slightly different covariates in Brazil. The first of these used temperature and precipitation with time lags of 3 months [28]. The second used an additional covariate ONI with time lags of 4 months [27]. The third used SOI with time lags of 6 months but precipitation and temperature were 1 and 2 months previously [26]. Lowe *et al*. [26] have highlighted that SOI significantly affects the time signal of dengue prediction. In southern Taiwan, it was found that the relative risk of DF increased when weekly minimum temperature increased with time lag over 4 weeks [49]. Therefore, in order to predict DF outbreak more precisely, a variety of temporal lags of climate variables and other covariates such as entomological data should be considered.

### Modelling approaches

Fully Bayesian methods are becoming more common as an alternative to the frequentist methods for spatial analysis of diseases. The benefit of Bayesian methods is that they can reduce the estimated variance particularly for regions with small populations [39]. Moreover, with Bayesian approaches, it is easier to incorporate variance components in a hierarchical manner and hence better estimate predictive uncertainty compared with frequentist methods based on maximum likelihood [26].

GLMMs also play a significant role in modelling spatial and spatio-temporal DF patterns. The inclusion of unstructured random effects in the model can account for overdispersion in dengue count distributions and allow for unknown factors. However, unstructured random effects are not able to overcome spatial dependence between locations. One way to allow for correlated heterogeneity between locations is the inclusion of spatially structured random effects [26]. Where GLMMs with spatial random effects have been applied, most studies have modelled the spatial random effect using an ICAR prior and adopted a binary adjacency-based neighbourhood spatial weight matrices. An additional two studies used a proper CAR prior [52, 54]. However, specific areas like rural areas or areas without neighbours need to be investigated in order to enhance the correlation structure in the model. For example, distance-based weight matrices, may be preferable for investigating the effect of road travel or human mobility. Only one study has used different types of neighbourhood adjacency matrices, namely, binary, weighted by the length of the boundary and by boundary and barriers [43]. Since the significance of some covariates change with the use of different adjacency structures, these different types of neighbourhood structures need to be taken into consideration. The inclusion of the spatially structured component using a CAR prior often improves model fit [38]. However, the impact of using other smoothing priors has not been done and needs to be further investigated.

Some studies have included both spatial and temporal random effects in modelling DF [26, 27, 48]. These authors assigned an intrinsic CAR prior to the spatially structured random effects and the first order autoregressive (AR (1)) prior for temporally structured random effects. An AR (1) model assumes that the current value is only affected by the prior value of the previous time period or temporal stage. Carroll *et al*. [72] considered temporal structure with a CAR prior distribution which allows more flexible structured variation over time; this model was applied to melanoma data. To increase the model fit, other time-series components that describe seasonal patterns, moving averages, trends, first- or second-order random walks should be considered.

Among the selected studies, only one study used a GLMM with spatial, temporal and spatio-temporal random effects [29], and only one with an additional temporal covariate [28]. Wijayanti *et al*. [29] assumed an interaction of two spatially unstructured and temporally unstructured random effects, which means there was no spatial and temporal structure on the interaction. More reasonable space–time interactions can be considered and compared. For example, the combination of spatially unstructured and temporally structured components may be more appropriate when the time trends differ between areas but the spatial structure is similar. Other types of space–time interactions that can be considered are a combination of spatially structured and temporally unstructured [41], or a combination of spatially and temporally structured components [73]. Lowe *et al.* [27] applied a first-order autoregressive monthly effect for each region in the space–time interaction, which means that the temporal trends are different for each region point and only depend on one previous time lag, without any spatial structure. The limitation of this interaction model is that neighbours in space are not considered. By working with adjacency matrices, neighbourhoods can be defined for both time and space and incorporated in spatio-temporal autoregressive (STAR) models [74].

Transmission models have been proposed by two studies [45, 54]. An SIR-SI included a transmission model for both the human and mosquito populations [54]. However, the mosquito population was assumed to be non-stochastic. Therefore, there needs to be better integration of transmission (mechanistic) and stochastic models. Other transmission models have been proposed by integrating four sub-models based on the Ross–Macdonald theory. This has been applied to a dengue study in China. However, all of the parameters for the independent variables were assumed to have normal distributions. Other prior distributions could be considered in order to detect patterns of dengue transmission more precisely.

### Study limitations

Only studies published in English were considered. It is acknowledged that there are other papers relevant to Bayesian spatial modelling in other languages, so we may have excluded valuable contributions. Furthermore, modelling dengue virus itself was not considered in this review, despite the acknowledged correlation between dengue virus and the number of dengue cases. Finally, inconsistencies in categorisation of some covariate variables, for example, indicators of socio-economic status, socio-demographics and environmental variables, have been found in a number of studies. These inconsistencies were not pursued here.

## Conclusions

Various Bayesian modelling approaches that aim to relate a range of possible explanatory variables with DF incidence or risk have been reviewed. Bayesian approaches are recommended instead of frequentist methods as they allow incorporation of a wider range of components of variance at different levels in the model and it is easier to obtain a more complete assessment of prediction uncertainty. Temperature and precipitation were important determinants of the relative risk of DF and predicting DF outbreak.

Most models used GLMM spatial random effects with spatially correlated effects using a CAR prior. Other GLMM models with the inclusion of temporal covariates and temporal trends were used to predict dengue risk. The only model that incorporated four sub-models, that is, female mosquito density dynamics, human daily movement, virus transmission and estimation of parameters was a transmission model based on Ross–Macdonald theory. To improve the precision of model fit of DF, different types of neighbourhood structure, proper and improper priors in spatial random effects, temporally structured effects and types of space–time interaction should be considered.

## Supplementary material

For supplementary material accompanying this paper visit https://doi.org/10.1017/S0950268818002807. click here to view supplementary material
