# Supplementary material for: Bayesian spatial and spatio-temporal approaches to modelling dengue fever: a systematic review
Source: Epidemiol Infect. 2018 Oct 29;147:e33. doi: 10.1017/S0950268818002807 (PMC6518570; doi:10.1017/S0950268818002807)
Supplement: Supplementary file 1 [file S0950268818002807sup001.docx]

**Epidemiology and Infection**

**Bayesian spatial and spatio-temporal approaches to modelling dengue fever, a systematic review**

A. Aswi, S.M. Cramb, P. Moraga and K. Mengersen

**SUPPLEMENTARY MATERIAL**

This file contains,

- Supplementary Table S1, Risk of bias tool for assessment
- Supplementary Table S2, Detailed content of studies using direct extraction from the publications
- Supplementary Table S3, Analytical method used in papers reviewed

**Supplementary Table S1, Risk of bias tool for assessment**

|  | **Criterion^[[1]](#footnote-1)^** | **Considerations^1^** | **Score considerations (0, none, 1, poor, 2, good)** |  |
| --- | --- | --- | --- | --- |
|  | **(A) Screening questions** |  |  | Definition,  Max 4 points |
| 1 | Does the paper clearly address aims and objectives? | Is the paper relevant to the objectives of the systematic review of Bayesian modelling? | 0 not stated  1 stated but vague  2 stated and focussed |  |
| 2 | Is the setting and population clearly defined? | Does the paper clearly state the setting (e.g. number of geographical location, number of dengue cases)? | 0 not stated  1 stated but vague  2 stated and focussed |  |
|  | **(B) Assessed the validity of model** | |  |  |
| 3 | Is the model structure clearly described and appropriate for the research question? | Is there a description of model structure (prior for space, time or space-time)?  Does the model structure include covariates? | 0 not appropriate model structure, or no description of model  1 incomplete description  2 complete description | Model methods, Max 4 points |
| 4 | Are the modelling methods appropriate for the research question? | Were the modelling methods clearly described, and suited to the research question? | 0 not appropriate modelling method, or no description of method  1 incomplete description  2 complete description |  |
| 5 | Are the parameters, ranges and data source specified? | Are all parameters and their ranges reported? | 0 poorly reported  1 some information missing  2 complete reporting of parameters, ranges and data sources | Model inputs, Max 4 points |
|  |  | Are the data sources for parameters reported? |  |  |
| 6 | Is the quality of data considered? | Are data limitations discussed? | 0 no sources of uncertainty  1 partially addressed, and/or data inappropriate  2 fully addressed |  |
|  | **(C) Assessed the overall results and study conclusion** | |  |  |
| 7 | Have the results been clearly and completely presented? | Do the results match the aims and objectives? | 0 not reported, very unclear  1 stated, but not directly aligned with research question  2 valuable and aligned with research question | Results, Max 4 points |
| 8 | Are the results appropriately interpreted and discussed in context? | Are the results of the study discussed in context and generalisability considered? | 0 no discussion  1 some discussion but key points and/or limitations missed  2 full discussion of key points, limitations discussed |  |

Total score, Max 16

Very high > 13

High 11-13

Medium 8 -10

Low < 8

(1) **Fone D, et al.** Systematic review of the use and value of computer simulation modelling in population health and health care delivery. *Journal of Public Health* 2003; **25**, 325-335.

(2) **Harris RC, et al.** Systematic review of mathematical models exploring the epidemiological impact of future TB vaccines. *Human Vaccines & Immunotherapeutics* 2016; **12**, 2813-2832.

**Supplementary Table S2, Detailed content of studies using direct extraction from the publications (abbreviations are described at the end of the table)**

| **Papers** | **References** | **Publication Year** | **Study area (period)** | **Dengue data** | **Covariate data** | **Objectives** | **Analytical method** | **Key finding** | **Further studies** | **Software** |
| --- | --- | --- | --- | --- | --- | --- | --- | --- | --- | --- |
| 1 | Astutik et al. | 2013 | East Java, Indonesia (2002-2008) | Monthly dengue cases in 38 districts | Monthly Rainfall index data (mm) considering spatial and temporal factors | To predict the association between the incidence of dengue endemic level and rainfall using Bayesian method | STARM | There is a positive correlation between the endemic level of DHF incidence and rainfall | Considering more independent variables and developing other models that can predict DHF incidence longer. | WinBUGS14 |
| 2 | Chien & Yu | 2014 | Southern Taiwan (1998 – 2011) | Weekly cases of dengue fever (742 weeks) in 107 districts. | Weekly meteorological data in every district (minimum temperature, and the maximum 24-hour rainfall) | To identify the relationship between the nonlinear delayed impact of meteorological variations and dengue risk and to predict dengue cases in the coming weeks. | A spatiotemporal quasi-Poisson model based on the DLNM approach | The most significant factors influenced dengue fever epidemic were the weekly minimum temperature and the maximum 24-hour rainfall. | Considering non-meteorological variables | R and SAS |
| 3 | Costa et al. | 2013 | Campinas, S$\tilde{a}$o Paulo State, Brazil (1 January to 31 December 2007) | Monthly dengue cases (11519 dengue cases) with 47 coverage areas | Socio-environmental deprivation strata, low deprivation, moderate and high deprivation. | To model the spatial distribution of dengue incidence and to investigate the relationship between the risk of dengue and socio-environmental deprivation strata | Bayesian hierarchical model. Dengue incidence data are assumed to be Poisson distributed | There is a positive correlation between spatial distribution of dengue risk and socio-environmental deprivation level in the age group older than 14 years, while in the 0 to 14 years age group, there is no correlation. | Additional information like vector ecology, circulating serotypes, and herd immunity is needed in interpreting the effects of socio-environmental deprivation strata to the relative risk of dengue. | R-INLA |
| 4 | Fernandes et al. | 2009 | Rio de Janeiro, Brazil (14 January 2001- 20 June 2002) | Weekly cases of dengue fever (77 weeks) with 156 districts | Levels of rainfall | To propose zero-inflated spatio-temporal model that can be applied for both continuous and discrete | Zero-inflated spatio-temporal model. | The proposed model can be used to estimate the probability of the presence of dengue disease that was not observed at region *i* and time *j.* | Considering other covariates, e.g. temperature | It is not mentioned |
| 5 | Ferreira &Schmidt | 2006 | Rio de Janeiro, Brazil (December 2001-May 2002) | Weekly number of dengue fever cases (25 weeks) for 156 districts. 125368 dengue fever notifications | Population, socioeconomic and geographical factors | To investigate the relationship between socioeconomic and geographical factors and the relative risk of dengue fever by considering three types of neighbourhood matrix structures | Bayesian spatial CAR model | The use of different adjacency structure caused changes to the significance of some covariate.  There is a negative correlation between slum areas and the relative risk of dengue | Considering a zero-inflated spatio-temporal model | WinBUGS |
| 6 | Honorato et al. | 2014 | Esp$\acute{i}$rito Santo, Brazil, in 2010 | 21933 cases of dengue with 78 municipalities | Sociodemographic variables, trash, water, literacy, income | To investigate the association between the risk of dengue fever and sociodemographic factors | Bayesian Spatial CAR model | Inadequate garbage disposal and income were the most significant factors related to the incidence of dengue | Using a set of data for many years | WinBUGS and R |
| 7 | Hu et al. | 2011 | Queensland, Australia (1 January 1993-31 December 2004) | Monthly notified dengue cases (12 years) | Geographic variation, (longitude and latitude) | To show the spatial distribution of dengue.  To identify spatial clusters and examine the spatio-temporal patterns of dengue spread | Spatial Empirical Bayes smoothing, LISA and spatio-temporal analysis methods | High incidence clusters were situated in Northern Queensland and low-incidence clusters were concentrated in the South-East Queensland | Determining the impact of socio-ecological factors (e.g. social, demographic, climate, vegetation, and mosquito density | GeoDa |
| 8 | Hu et al. | 2012 | Queensland, Australia (1 January 2002-31 December 2005) | Monthly numbers of locally acquired and overseas-acquired DF cases across 125 LGA | Data on weather (temperature and rainfall), SEIFA, sociodemographic factors (population size) and number of overseas travellers | To quantify the relationship between DF and socioecological factors and to evaluate differences for local and overseas spatial patterns and predictors. | Bayesian Spatial CAR model. | The spread of DF is influenced by socioecological factors. The drivers seem different for local and overseas cases. Spatial clustering of dengue cases was obvious. | Considering other factors such as mosquito, Human behaviours, and population immunity that may affect the spread of dengue | WinBUGS |
| 9 | Jaya et al. | 2016 | Bandung, Indonesia (2012) | Dengue fever cases (5095 people) with 30 sub-districts | Population density, larva-free home index, healthy housing index, and rainfall. | To compare between FCM and VCM in explaining the environmental factors that influence the number of DF diagnoses | Bayesian spatial CAR models, FCM and VCM | VCM model estimates relative risk better than FCM model.  The most significant effect on the relative risk of dengue fever is a larva-free home. | Developing a spatio-temporal lag model and including climate factors into the model | R-INLA |
| 10 | Johansson et al. | 2009 | Puerto Rico (July 1986 –December 2006) | Monthly dengue incidence for 20 year period) with 77 municipalities | Monthly mean maximum temperature, mean minimum temperature, and cumulative precipitation | To analyse the relationship between monthly change in temperature and precipitation and monthly changes in the spread of dengue | Hierarchical model with adaptive natural cubic spline | The positive correlation between monthly variation in temperature and precipitation and the spread of dengue is statistically significant and this correlation varies spatially | Evaluating the correlation between multi-year climate differences and dengue cases. | Tlnise  R |
| 11 | Kikuti et al. | 2015 | Salvador, Brazil (1 January 2009- 31 December 2010) | Laboratory-confirmed dengue during the year study period is 651 with 98 census tracts | Demographic, socioeconomic, geographical data | To assess the association between covariate variables (demographic, socioeconomic and geographic) and dengue risk in urban slum. | Poisson log-normal model and CAR model (spatial) | Lower socioeconomic status (within slum society) influenced the increases risk of dengue. They also found that, model fit increased when a spatially structured term was included | Considering the distance between health service facilities and each area when assessing risk | R INLA |
| 12 | Lekdee & Ingsrisawang | 2013 | Northern Thailand (2011) | Dengue data consisted of 68 observations (4 quarterly observations) from 17 provinces | Climate, rainfall, temperature | To develop a disease map and analyse the association between dengue data, rainfall and temperature | GLMMs with proper CAR  Spatial random effects. The numbers of dengue fever patients are assumed to be Poisson distributed. | There was a significant positive correlation between Relative Risk of dengue fever, rainfall and temperature.  DF maps can precisely show the high risk areas. | - | Open BUGS |
| 13 | Lowe et al. | 2011 | South East region of Brazil (January 2001- December 2008) | Monthly cases of dengue fever (96 months), over 558 microregions | Temperature, precipitation, an ENSO Index and altitude, and percentage of urban population | To assess the inclusion of climate covariates, seasonal climate forecast and random effects in the model to provide early warnings | Spatio-temporal GLM and GLMM | The inclusion of random effects (GLMM model) to the model has enhanced dengue predictions and this model can be used to forecast dengue incidence in advance. | Understanding the correlation of temperature and precipitation patterns with warm phase and cold phase ENSO for this region  -considering nonlinear climate variable | WinBUGS |
| 14 | Lowe et al. | 2013 | Southeast Brazil (January 2001 to December 2009) | All notified dengue cases from hospitals and clinic doctors (monthly dengue cases = 108 months) with 160 microregions | Non-climate factors, cartographic, demographic, and socio-economic variables.  Climate factors, temperature, precipitation, and the ONI | To test whether the extended model can predict dengue risk better than a simple model | A negative binomial GLMM | The extended model which consists of climate variables, past dengue risk, spatially structured and unstructured random effects and temporal random effects improved dengue predictions. | Considering entomological data, seasonal movement of human hosts.  A proximity matrix used in CAR prior based on the hierarchical matrix instead of on its neighbour | R, WinBUGS |
| 15 | Lowe et al. | 2014 | Brazil (January 2000-December 2013) | Monthly dengue cases (168 months) with 553 microregions | Precipitation, temperature anomalies, altitude, population density and the log ratio of observed to expected dengue relative risk 4 months ahead | To predict dengue epidemic during the tournament in Brazil | A Bayesian GLMM | The model can forecast which cities have low-, medium- and high- risk of dengue | - | R, WinBUGS |
| 16 | Lowe et al. | 2016 | Thailand (1982-2013) | Monthly severe cases (DF, DHF, DSS) for 384 months in the 76 provinces of Thailand | Demography data, Meteorological data (Precipitation and temperature data) | To measure the added value of including climate functions | GLMM / GAMM using a negative binomial likelihood. | Model with the inclusion of linear and nonlinear functions of climate explained 39% and 40% respectively of the variation in dengue relative risk. There is an additional value 7 % and 8% to the variation explained by seasonal-spatial structure | Find out the reason why the correlation between relative risk of dengue and precipitation 4–6 months formerly was negative. | R-INLA |
| 17 | Martı´nez-Bello et al. | 2018 | Colombia (January 2009 –December 2015) | 25365 dengue cases (dengue and severe dengue)  293 census sections  91 epidemiological periods (EP) | Land surface temperature (LST)  Normalized difference vegetation index (NDVI) | To explore type I-IV interaction effect spatiotemporal models of relative risk of dengue disease  To assess the association between covariate variables and dengue disease | Bayesian Poisson spatiotemporal interaction effect models | The best model was model with the inclusion of a fixed coefficient of lag-zero epidemiological periods (EP) LST and type IV interaction effects.  There was a weak positive correlation between LST and cases of dengue by census section | Inclusion data with small resolution in space and time.  Considering other covariates like rainfall and humidity.  Integration spatiotemporal relative risk mapping of dengue to online platform.  Fitting spatiotemporal relive risk models with at least one level two levels of aggregations. | WinBUGS and OpenBUGS |
| 18 | Martı´nez-Bello et al. | 2017 | Colombia (January 2008 –December 2015) | 27301 cases  293  census sections , 1-433 | Land surface temperature (LST)  Normalized difference vegetation index (NDVI) | To compare convolution models with CAR priors and Leroux CAR priors for estimating relative risk of dengue | Bayesian hierarchical Poisson model using ICAR prior and Leroux CAR prior for spatially structured random effects | For estimating relative risk, NDVI provide more information than LST.  There was a positive correlation between NDVI and relative risk of dengue.  Convolution model with CAR priors was better than Leroux CAR priors in fitting the data | Applying this Hierarchical Bayesian models in mapping the relative risk of other arboviral diseases | WinBUGS and  R |
| 19 | Mukhsar et al. | 2016a | Kendari,, Southeast Sulawesi, Indonesia (2007-2013) | Monthly DHF cases (84 months) for ten sub-districts | Rainfall and population density | To extend the convolution model proposed by Sani et al. by including the probability of incident risk | Bayesian spatio-temporal model based on an  extended convolution model | The extended relative risk model is more sufficient precision compared to model developed by Ecker et al. and Sani et al.  Both rainfall and population density are associated with increases in dengue cases. | Considering zero-inflated Poisson spatio-temporal model | WinBUGS |
| 20 | Mukhsar et al. | 2016b | Kendari, Southeast Sulawesi Province, Indonesia (2013-2015) | Monthly DHF cases (2064 cases) in ten districts | - | To construct the posterior distribution of the BMZIP S-T | A BMZIP S-T model | They have been constructing the posterior distribution of BMZIP S-T model | Finding a full conditional distribution of the model based on the posterior distribution. | Not mentioned |
| 21 | Pepin et al. | 2015 | Vitoria city, Brazil (November 2007-December 2011) | Weekly dengue fever cases | Vector density, lagged case data and spatial connectivity | To assess the role of global vector data in forecasting dengue fever | GLMM with a Poisson error structure | Global between-neighbourhood effect (city-wide) were more robust than within-neighbourhood or nearest-neighbourhood effects. | Determining which data could best describe city- wide connectivity | R-INLA.  ArcGIS |
| 22 | Restrepo et al. | 2014 | Colombia (1 January 2007- 31 December 2010) | Monthly number dengue cases (a total of 304984 dengue cases) with 1124 municipalities, but only include1065 municipalities | Environmental factors, temperature, precipitation and elevation | To determine the spatial distribution of dengue cases and the associations between environmental factors and the dynamics of the disease. | A Bayesian spatio-temporal CAR model | The distribution of dengue cases in the eastern and central part of Colombia is spatially clustered.  The most significant predictor of dengue transmission is precipitation | Should investigate the role of ENSO | WinBUGS |
| 23 | Samat & Percy | 2012 | Malaysia (2008-2009) | Weekly dengue cases (week 1 to 53) with 16 states | - | To develop an alternative method for estimating the relative risk of dengue | Discrete Time-Space stochastic SIR-SI models | Putrajaya is categorized as a very high risk for dengue occurrences, and Selangor and Kuala Lumpur are categorized as high risk | Considering other informative priors and their impact.  Using continuous time and discrete space | WinBUGS |
| 24 | Sani et al. | 2015 | Kendari,, Southeast Sulawesi, Indonesia (2007–2010) | Monthly DHF cases (48 months) for ten sub-districts | Rainfall and population density | To develop a spatio-temporal convolution model and to analyse the relationship between rainfall, the population density and the risk of dengue cases. | A spatial–temporal convolution (Poisson-lognormal) model with spatio-temporal random effects | Their model can be used to determine when and where the dengue incidence may occur.  Both rainfall and population density increased the risk of dengue cases. | Considering other non-dynamic relevant covariates such as ponds, reservoirs, and bushes and the extending the model to a zero-inflated model | WinBUGS |
| 25 | Vargas et al. | 2015 | Itabora$\acute{i}$municipality, Rio de Janeiro State, Brazil (2007-2008) | Monthly dengue incidence (total of 4281 notified cases) with eigth districts (79 neighbourhoods) | Entomologic data (the home infestation index), sociodemographic data | To identify spread areas of dengue by looking for the relationship between HI, dengue incidence, and sociodemographic factors. | Local Empirical Bayes approach.  Kernel density estimate with the kernel quartic function is used to detect the higher risk areas for dengue occurrence. | There is a positive correlation between HI and dengue incidence rate.  The highest dengue risk regions were situated in areas which had the highest population densities and were close to major highways. | To avoid over-estimation, the data related to disease should be recorded by the probable infection locale instead of by residence address. | ArcGIS 10.0 |
| 26 | Vazquez-Prokopec et al. | 2010 | Cairns, north-eastern, Queensland, Australia (January –August 2003) | Weekly dengue infection (25 weeks) with 383 laboratory-confirmed human dengue cases and 1490 premises | Distance to the epidemic’s index case (IRS), rain, and non-linear temporal trend, temperature (mean, min, and max) | To assess the impact of rain, spray cumulative proportion and spatial autocorrelation on the odds of weekly dengue virus infection | A semi-parametric Bayesian space-time STAR model | There was a significant positive correlation between the number of IRS applications up to a time lag of 2 weeks and the weekly number of cases. | Applying this model to other areas with dengue | ArcGIS, BayesX, and R |
| 27 | Wijayanti et al. | 2016 | Banyumas regency, Central Java, Indonesia (1 January 2000-31 December 2013) | All hospital-reported dengue cases with 329 villages | Population and socio-economic data (age, working status and education level for each village), Environmental data (EVI, day-time (LST) and nLST, precipitations) | to determine factors influencing the risk of dengue | A Bayesian Poisson model including spatial, temporal and spatio-temporal random effects. | The most significant factors that influence the risk of dengue are employment type and the education level | Factor variations in immunity need to be investigated. | R-INLA |
| 28 | Yu et al. | 2011 | Southern Taiwan (2002-2007) | Weekly dengue incidence (318 weeks) in 108 townships | Climatic variables, i.e. weekly average/maximum/minimum temperature, logarithm of rainfall, SOI, Breteau Index. | To examine the association between DF incidence and the selected climate variables.  To predict the DF outbreaks based on place and time. | A spatio-temporal stochastic BME method | Climatic conditions significantly affect DF outbreaks.  The model can predict DF outbreak fairly accurately 1 week in advance. | Incorporating spatial information sources (e.g. land use, remote sensing data, vegetation cover indicators) | Quantum GIS (QGIS) |
| 29 | Yu et al. | 2014 | Kaohsiung, Taiwan (2002) | Weekly cases of DF infection in 11 districts | - | To obtain online space-time predictions of DF transmission | BME-SIR model. | The BME-SIR is able to predict precisely space-time DF spread | Investigation of other covariate variables such as mosquito population, human interventions, the size of residential areas, water bodies, and farmlands | BMElib |
| 30 | Yu et al. | 2016 | Southern Taiwan (1998-2012) | Annual dengue cases of 107 districts | Hydrological factors, average, the maximum, the minimum of temperature; the total, the maximum 24-h and them maximum 1-h of  Rainfall | To develop an EWS model by considering hydrological factors based on space and time | BME method | Weekly maximum 24-h rainfall and weekly minimum temperature are significantly affect the dengue fever occurrence.  Early warning of DF can be provided by the spatiotemporal predictions | Investigation of other covariate variables such as land use and imported case data | Not mentioned |
| 31 | Zhu et al. | 2016 | Guangzhou, China (September to November 2014) | Daily dengue cases in 12 districts | Entomologic, demographic, and environmental data | To identify the spatio-temporal transmission patterns of dengue epidemics. | A dengue transmission model based on Ross-Macdonald theory | Urban areas have the highest incidence rates and suburban areas have the second highest incidence rates. | The proposed models need to be improved. | Not mentioned |

**Abbreviations used,**

SOI, Southern oscillation index; BI, Breteau index; HI, house index; DF, dengue fever; BME, Bayesian maximum entropy; SIR, susceptible-infected-recovered; CAR, conditional autoregressive; LGA, local government area; SEIFA, socioeconomic index for areas; DHF, dengue hemorrhagic fever; DSS, dengue shock syndrome; GLMM, generalized linear mixed model; GAMM, generalized additive mixed model; ENSO, El-Nino-Southern Oscillation; STARM, spatial-temporal autologistic regression model; DLNM, the distributed lag non-linear model; EVI, enhanced vegetation index; nLST, night-time land surface temperatures; IRS, indoor residual spraying; STAR, structured additive regression; FCM, fixed coefficient model; VCM, spatially varying coefficient model; BMZIP S-T, Bayesian mixture zero inflated Poisson spatio-temporal; SIR-SI, susceptible-infective-recovered for human populations - susceptible-infective for mosquito populations, LISA, local indicators of spatial association

**Supplementary Table S3, Analytical method used in papers reviewed**

| **Paper** | 1 | 2 | 3 | 4 | 5 | 6 | 7 | 8 | 9 | 10 | 11 | 12 | 13 | 14 | 15 | 16 | 17 | 18 | 19 | 20 | 21 | 22 | 23 | 24 | 25 | 26 | 27 | 28 | 29 | 30 | 31 | Total |
| --- | --- | --- | --- | --- | --- | --- | --- | --- | --- | --- | --- | --- | --- | --- | --- | --- | --- | --- | --- | --- | --- | --- | --- | --- | --- | --- | --- | --- | --- | --- | --- | --- |
| **Spatial Models** |  |  |  |  |  |  |  |  |  |  |  |  |  |  |  |  |  |  |  |  |  |  |  |  |  |  |  |  |  |  |  |  |
| - Empirical Bayes |  |  |  |  |  |  | ✓ |  |  |  |  |  |  |  |  |  |  |  |  |  |  |  |  |  | ✓ |  |  |  |  |  |  | 2 |
| - GLMM with spatial random effects |  |  | ✓ |  | ✓ | ✓ |  | ✓ | ✓ |  | ✓ |  |  |  |  |  |  | ✓ |  |  |  |  |  |  |  |  |  |  |  |  |  | 7 |
| **Spatio-temporal models** |  |  |  |  |  |  |  |  |  |  |  |  |  |  |  |  |  |  |  |  |  |  |  |  |  |  |  |  |  |  |  |  |
| - GLMM with spatial random effects |  |  |  |  |  |  |  |  |  |  |  | ✓ |  |  |  |  |  |  |  |  |  |  |  |  |  |  |  |  |  |  |  | 1 |
| - GLMM with spatial random effects +temporal covariate |  |  |  |  |  |  |  |  |  |  |  |  |  |  |  |  |  |  |  |  | ✓ | ✓ |  |  |  |  |  |  |  |  |  | 2 |
| - GLMM with spatial and temporal random effects +temporal covariate |  |  |  |  |  |  |  |  |  |  |  |  | ✓ | ✓ |  | ✓ |  |  |  |  |  |  |  |  |  |  |  |  |  |  |  | 3 |
| - GLMM with spatial, temporal and spatio-temporal random effects +temporal covariate |  |  |  |  |  |  |  |  |  |  |  |  |  |  | ✓ |  |  |  |  |  |  |  |  |  |  |  |  |  |  |  |  | 1 |
| - GLMM with spatial, temporal and spatio-temporal random effects |  |  |  |  |  |  |  |  |  |  |  |  |  |  |  |  | ✓ |  |  |  |  |  |  |  |  |  | ✓ |  |  |  |  | 2 |
| - GLMM with spatio-temporal random effects+temporal trend |  |  |  |  |  |  |  |  |  |  |  |  |  |  |  |  |  |  | ✓ |  |  |  |  | ✓ |  |  |  |  |  |  |  | 2 |
| - GLMM zero-inflated Poisson |  |  |  | ✓ |  |  |  |  |  |  |  |  |  |  |  |  |  |  |  | ✓ |  |  |  |  |  |  |  |  |  |  |  | 2 |
| - A spatio-temporal quasi-Poisson model |  | ✓ |  |  |  |  |  |  |  |  |  |  |  |  |  |  |  |  |  |  |  |  |  |  |  |  |  |  |  |  |  | 1 |
| - Hierarchical model with adaptive natural cubic spline |  |  |  |  |  |  |  |  |  | ✓ |  |  |  |  |  |  |  |  |  |  |  |  |  |  |  |  |  |  |  |  |  | 1 |
| - BME |  |  |  |  |  |  |  |  |  |  |  |  |  |  |  |  |  |  |  |  |  |  |  |  |  |  |  | ✓ | ✓ | ✓ |  | 3 |
| - SIR-SI |  |  |  |  |  |  |  |  |  |  |  |  |  |  |  |  |  |  |  |  |  |  | ✓ |  |  |  |  |  |  |  |  | 1 |
| - STARM | ✓ |  |  |  |  |  |  |  |  |  |  |  |  |  |  |  |  |  |  |  |  |  |  |  |  |  |  |  |  |  |  | 1 |
| - Semiparametric Bayesian STAR |  |  |  |  |  |  |  |  |  |  |  |  |  |  |  |  |  |  |  |  |  |  |  |  |  | ✓ |  |  |  |  |  | 1 |
| - Ross-Macdonald transmission |  |  |  |  |  |  |  |  |  |  |  |  |  |  |  |  |  |  |  |  |  |  |  |  |  |  |  |  |  |  | ✓ | 1 |
| **Total** |  |  |  |  |  |  |  |  |  |  |  |  |  |  |  |  |  |  |  |  |  |  |  |  |  |  |  |  |  |  |  | **31** |

1. Adapted from Fone et al. (1) and Harris et al. (2). [↑](#footnote-ref-1)
